# Cholangiocarcinoma Disease Modelling Through Patients Derived Organoids

**DOI:** 10.3390/cells9040832

**Published:** 2020-03-30

**Authors:** Francesco Amato, Colin Rae, Maria Giuseppina Prete, Chiara Braconi

**Affiliations:** 1 Institute of Cancer Sciences, University of Glasgow, Glasgow G61 1BD, UK; 2496589A@student.gla.ac.uk (F.A.); Colin.Rae@glasgow.ac.uk (C.R.); MariaGiuseppina.Prete@glasgow.ac.uk (M.G.P.); 2 Humanitas Cancer Center, Humanitas Clinical & Research Center, IRCCS, Rozzano, 20089 Milan, Italy; 3 Beatson West of Scotland Cancer Centre, Glasgow G12 0YN, UK

**Keywords:** CCA, organoid, PDO, personalized medicine, biliary cancer

## Abstract

Cancer organoids are 3D phenotypic cultures that can be established from resected or biopsy tumour samples and can be grown as mini tumours in the dish. Flourishing evidence supports the feasibility of patient derived organoids (PDO) from a number of solid tumours. Evidence for cholangiocarcinoma (CCA) PDO is still sparse but growing. CCA PDO lines have been established from resected early stage disease, advanced cancers and highly chemorefractory tumours. Cancer PDO was shown to recapitulate the 3D morphology, genomic landscape and transcriptomic profile of the source counterpart. They proved to be a valued model for drug discovery and sensitivity testing, and they showed to mimic the drug response observed in vivo in the patients. However, PDO lack representation of the intratumour heterogeneity and the tumour-stroma interaction. The efficiency rate of CCA PDO within the three different subtypes, intrahepatic, perihilar and distal, is still to be explored. In this manuscript we will review evidence for CCA PDO highlighting advantages and limitations of this novel disease model.

## 1. Introduction

**Figure 1:**
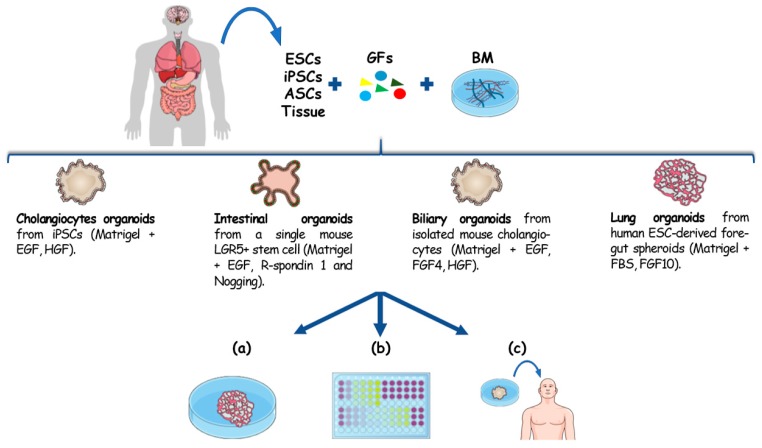
Organoids establishment from different cell sources and their downstream applications. Organoids can be originated from different sources: ESCs, ASCs, iPSCs and primary tissue. The embedding of these cells in a basal membrane (BM), mostly represented by Matrigel, with appropriate growth factors has been reported to give rise to cholangiocytes [9] intestinal [3], hepatobiliary [10] organoids and lung [7]. For the capability to recapitulate 3D architecture, genotype and the histomorphology of the in vivo counterpart these constructs can be exploited for (**a**) disease modelling, (**b**) drug screening and precision medicine. Given their genome stability and the possibility to establish an organotypic culture with patient cells, organoids also showed to be a promising tissue source for regenerative medicine (**c**).

Organoids are microscopic three-dimensional (3D) tissue-like structures that outgrow from primary tissue explants or from single cells. Advances in 3D culturing techniques enabled the expansion of single stem cells into self-organizing tissues that functionally recapitulate key aspects of their in vivo tissue of origin [1]. Stem cell organoids can mimic the stem cell niche and can be derived from embryonic stem cells (ESCs), induced pluripotent stem cells (iPSCs) and tissue resident adult stem cells (ASCs) Figure 1. While ESC- and iPSC-derived organoids recapitulate embryonic developmental processes, organoids derived from ASCs represent a diversity of organotypic cultured tissues [1,2], where tissue homeostasis, or its disruption during disease, is recapitulated. This technology is commonly based on the use of 3D culture systems characterized by the presence of a basal membrane, acting as an extracellular matrix (ECM), and growth factors. Once embedded in ECM, these cells self-assemble and differentiate in response to physical and molecular cues giving rise to 3D constructs that genotypically and phenotypically recapitulate the in vivo counterpart. The work that opened the avenue to organotypic culture was published in 2009 by Sato et al. [3]. Through this study they explored how the combination of a laminin-rich basal membrane (Matrigel) and a cocktail of growth factors (including the Epidermal Growth factor, R-spondin 1 and Noggin) gave rise to gut epithelial organotypic constructs with villus-crypt structures starting from a single mouse Lgr5+ intestinal stem cell [3]. Since then, a plethora of different human and mouse organoid cultures have been established [4,5,6,7,8], and are now used as 3D ex vivo models of a number of organs and diseases.

## 2. Hepatobiliary Organoids

During the last decade it has been possible to observe an exponential growth in organoid research. Nevertheless, liver organoids remain poorly represented compared to organotypic constructs resembling other organs. Despite this, first attempts to create 3D liver tissues had already been reported at the beginning of this century. Particularly, Michalopoulos et al. were able to bring more insights about the tissue organization of liver through a culture system characterized by roller bottles whose internal surfaces were covered with collagen [11]. Isolating hepatocytes from rat liver though the collagenase perfusion technique and culturing them for 20 days with a medium including hepatocyte growth factor (HGF), epidermal growth factor (EGF) and dexamethasone they observed a 3D sheets-like construct with the surface facing the medium characterized by a biliary epithelium, and the layer below comprising of hepatocytes embedded in connective tissue and in contact with a layer of endothelial cells. Through this approach they reported how the dexamethasone was essential for the maturation of hepatocytes, since the absence of this molecule in culture caused a reduction of up to 15% of immature Hepar+ and cytochrome *p*-450+ hepatocytes; conversely, the combination of HGF and EGF was essential for the maturation of the biliary epithelium and for the development of the connective tissue. Likewise, Doffou et al. showed the role of other genes, such as OCT4, in the hepatocyte-biliary transdifferentiation. Inhibition of this factor induced the incapacity of biliary cells facing the medium to complete their transition from hepatocytes to biliary cells [12]. To date, it is still elusive of which cells in the liver serve as the cell-of-origin for the organoids. Animal experiments showed that when all liver tissue was dissociated and cultured in Matrigel, there was a preponderance of cholangiocytes markers (cytokeratin 19, CK19) over hepatocyte markers (albumin, ALB). Likewise, deprivation of Noggin and Jagged-1 in the media for organoid propagation induced a higher ratio of CK19/ALB, suggesting that progenitor cells might commit to biliary differentiation under 3D culture [13]. The theory of cell plasticity is also confirmed by the evidence that an organoid line established from an intrahepatic cholangiocarcinoma (iCCA) patient-derived xenograft (PDX) displayed a hepatocyte-like phenotype when culture with the appropriate differentiation medium used for human liver organoids. Wnt pathway deregulation and epigenetic control seemed to be the driving forces for the switch from iCCA to the hepatocyte phenotype [14]. Similarly, spheroids from human induced hepatocytes (generated from umbilical cord fibroblasts) could be differentiated into hepatocellular carcinoma (HCC) through Myc activation or into iCCA thorugh Kirsten Rat Sarcoma (KRAS) activation [15]. The development of 3D culture systems based on the use of Matrigel as artificial ECM brought more sophisticated studies on liver organogenesis. Wu et al. 2019 were able promote the development of hepatobiliary organoids starting from human induced pluripotent stem cells (hiPSCs) in 45 days [16]. Notch appears to act as a driving factor for the biliary morphogenesis [16,17]. Addition of a NOTCH inhibitor in the culture medium led to the total absence of mature ductal structures, with a significant decrease of transcription factors regulating cholangiocytes’ differentiation, such as HNF6 and Sox9 [17]. Genome stability and maintenance of tissue identity over long term expansion opened the possibility of applying hepatobiliary organoids in regenerative medicine [18,19]. Huch et al. reported an increased genome stability in ASC derived organoids compared to organoids derived from reprogrammed iPSCs [18]. Normal liver ASC derived organoids accumulate between 63 and 139 base substitution per culture, which appears to be 10-fold less compared to the liver iPSC-derived organoids [18,20]. Sampaziotis et al. demonstrated how extrahepatic cholangiocytes organoids continued to express biliary markers in 98% of cases after 20 passages, while they also maintain their functional extrusion capabilities [19]. Different attempts to apply this technology in vivo have been reported. Hu et al. showed that human hepatocyte-derived organoids engrafted in immune compromised mice with damaged liver can give rise to repopulation and proliferation over a period of 90 days with final cell population expressing functional maturity markers ALB, MRP2 and CYP2E1 [21]. Sampaziotis et al. cultured human primary extrahepatic cholangiocytes’ organoids (ECO) and showed that, when seeded in a biocompatible scaffold, were able to reconstruct an injured tract of the biliary tree in vivo [19]. Although more work needs to be carried out, these studies could open a new avenue to develop novel applications in regenerative medicine. Since the cells are established directly from the donor, these methods have the potential to avoid possible rejections in transplantation medicine.

## 3. Cancer Patient Derived Organoids

Cancer models are used in basic research to unveil mechanisms of cancer development and progression and to identify vulnerabilities that can be exploited for cancer therapeutics. Cancer cell lines (CCLs) derived from human cancer tissues have been widely used for many years to model cancer phenotypes in vitro. CCLs are grown as two-dimensional (2D) cultures and have advantages such as an ease of culture with the possibility to grow over time, a lack of need for elaborate media, fast growth rate and suitability to high-throughput drug screening and functional experiments. At the same time, this model has several limitations such as the low efficiency to establish cell lines from the origin tumour due to the reduced adaptation of cells in the new environment, and loss of identity over time with the potential accumulation of new molecular aberrations due to the proliferation capacity combined with several passages required in culture. Furthermore, CCLs do not recapitulate the conditions observed in vivo such as cell-to-cell interaction, cell-to-ECM interplay and tumour-to-microenvironment crosstalk that is known to be crucial for cell function and drug response. Tumour spheroids are 3D clusters of cells that form as the result of the tendency of adherent cells to aggregate when grown in suspension. They were demonstrated to be an improved model over CCLs to mimic drug response/resistance due to their potential to recapitulate cellular layered assembling, hypoxia and nutrient gradients [22]. However, they lack the histomorphology and 3D-architecture of human tumours. Animal models are more useful when a phenotype needs to be studied in vivo and the contribution of the microenvironment requires to be addressed. In an attempt to reproduce and conserve the patient tumour characteristics (such as heterogeneous histology, malignant phenotypes and genotypes, tumour architecture and vasculature) patient-derived xenografts (PDX) have been established [23,24]. PDX are models based on the engraftment of cancer cells or small pieces of tumour tissue orthotopically or subcutaneously in immunocompromised mice. After the engraftment, cancer cells or tissues undergo a vascularization process allowing a more physiological environment. This model has shown to be remarkably useful, especially for drug testing, because it allows researchers to mimic the response in vivo to a given drug. However, this model has several drawbacks: it lacks the immune system and the original surrounding stromal cells; it is time consuming, highly expensive and not suitable for high-throughput screening Table 1. Patient derived organoids (PDO) have filled the gap between CCLs/spheroids and PDX as powerful tools to study tumour morphology and functionality, while taking into consideration cell-to-cell and cell-to-ECM interactions, to allow systematic chemical and genetic perturbation combined with in toto imaging of 3D tumour tissues over time. Improvements of protocols for 3D organotypic cultures allowed researchers to obtain cancer organoids directly from patient biopsy material providing individualized cancer models that reproduce interpatient heterogeneity [25,26]. Histological analysis showed that these constructs retained the microscopic features and histomorphologic architecture observed in the tumour source. Similarly, molecular characterization confirmed that the mutational landscape of the original tumour is retained in the PDO [25,26].

## 4. Cholangiocarcinoma Patient-Derived Organoids (CCA PDO)

**Figure 2:**
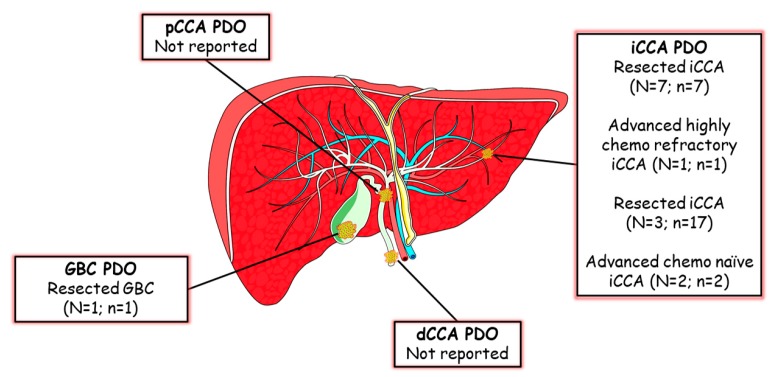
Patient derived organoid (PDO) lines from biliary tract malignancies. Graphic representation of four types of biliary tract malignancies with relative PDO lines generated so far for GBC [5] and iCCA (from top to bottom of the box [4,5,14], [32], [35], [36]). (N: number of patients; n: number of PDO lines).

Cholangiocarcinomas (CCAs) are aggressive and heterogeneous malignancies arising from the biliary tree and, based on the anatomical location, can be categorized in intrahepatic (iCCA), perihilar (pCCA) and distal (dCCA). Only 20% of patients undergo surgery, while the majority of CCA patients are diagnosed at an advanced stage. Evidence of CCA PDO is still sparse Figure 2. Broutier et al. established long term cultures of primary liver cancer organoids from treatment-naïve resected specimens of three subtypes of liver cancer [4]. As normal liver cell populations are known to give rise to liver organoids, the issue of normal liver outgrowth was targeted using a tumoroid-specific medium lacking R-sponding-1, Noggin and Wnt3a [18]. The efficiency of PDO establishment seemed to correlate with the proportion of proliferating cells requiring at least 5% proliferative index in the tumour sample. As seen for other tumour types, these PDO maintained histological features (with EpCam+ positivity for iCCA), expression profiles and marker expression of the parental tissue (keratin 7 for iCCA) after long term expansion in culture. Taking advantage of the whole cohort of primary liver cancers (HCC = 3; iCCA = 2 and mixed HCC/CCA = 2) and the cohort of normal liver organoids, Broutier et al. derived a liver tumoroid gene signature. Resected early stage liver cancers have been extensively studied and molecularly characterized [27,28,29,30,31]. However, gene expression profiles of tumour organoids allowed identification of a further 11 genes that had never previously been reported associated with liver cancer before and had prognostic value, with a specific value for C1QBP for iCCA. Given the tumoroids recapitulated the morphological and functional aspects of the source tissues, differences between HCC and iCCA were retained ex vivo at all levels. If PDO from resected specimens contribute to provide further disease models to investigate biology and test drug sensitivity, their application is limited in clinical practice as the majority of patients are diagnosed at an advanced stage. Lampis et al. provided the first evidence that PDO can be established from biopsies of advanced iCCA patients who failed more than one chemotherapy line [32]. The combination of PET-scan and CT-guided fine needle biopsy was used to identify the tumour area responsible for disease progression, thus generating a model of highly chemoresistant CCA. Not only the PDO did retain the morphological architecture of human CCA with glandular domain, and genomic profiles of the source tissue, but also displayed a similar drug-resistance in line with the chemoresistance shown in the patient, thus offering a clinically relevant platform for drug discovery. Indeed, these PDO were used to validate data from drug discovery projects providing an additional layer in the preclinical assessment of novel therapeutics. In addition, PDO have the advantage of being a flexible tool where drug efficacy can be tested in association with biomarker development through PDO genomic manipulation that is feasible and effective [32]. Lampis et al. were able to show a positive correlation between MIR21 expression and resistance to Heat Shock Protein 90 inhibitors via a controlled lentiviral transcriptional activation of the miRNA ex vivo (PDO) and in vivo (PDO-derived PDX) [32]. Similarly, Artegiani et al. have used genomic manipulation of normal cholangiocytes’ organoids to recreate the genetic background of CCA. Electroporation protocols enabled transfection of CRISPR/Cas9 vectors inducing frameshift mutations in both alleles of the BAP1 gene. The 3D nature of organoids allowed identification of the role of BAP1 in maintenance of the epithelium integrity and cell polarity, which was also confirmed in orthotopic liver transplantation of genetically engineered organoid lines [33]. Similarly, genetic manipulation of murine liver and gallbladder organoids allowed the investigation of the oncogenic potential of a number of molecular aberrations, showing that *KRAS* may have a role in tumour initiation, while other alterations, such as PIK3CA mutation and FGFR fusions, occur later in tumour progression and have less tumorigenic potential [13,34]. Saito et al. derived four biliary cancer patient PDOs, including three iCCAs and one gallbladder cancer (GBC), all established from early stage resected tumours [5]. Interestingly, they were able to show that the growth kinetics differ between organoids derived from tumour and normal tissue. While cancer organoids can be kept in culture for a long time (>1 year) and easily expanded after freezing without morphological changes, organoids derived from non-cancer gallbladder and bile duct tissues showed a higher proliferation rate at the early passages but ceased to proliferate after passage 15. As expected, the genomic profile of CCA PDO were different from non-cancer bile duct organoids, in terms of both the coding and non-coding transcriptome [5].

## 5. Applications of Patient Derived Organoids in CCA

CCA PDO recapitulate the 3D architecture, morphology, genotype and phenotype of human CCA, while they can be easily manipulated and as such, they provide a valuable tool for functional characterization of CCA behaviour. Compared to animal models they are faster and cheaper offering the opportunity to create a large biobank of PDO lines that can be used to reproduce ex vivo the heterogeneity of CCA subtypes. Nonetheless, PDO have several limitations in comparison to animal models, which we will address in the next chapter. Thus, it is likely that for an appropriate expansion of CCA knowledge a combination of several models will be more useful than relying on one specific model. As described earlier, PDO represent a relevant model to assess drug response and promote a drug discovery project associated to biomarker development. Novel genes have been identified to maintain CCA growth over normal counterpart, suggesting that PDO can help in the identification of novel targets for therapeutic development that potentially target the 3D growth of CCA and the cell-to-cell and the cell-to-ECM interactions [4]. Furthermore, this technology is amenable to identify markers associated with drug sensitivity/resistance and to carry out de-novo high-throughput drug screening to identify new effective drugs with reduced adverse side effects. Saito et al. and Van de Wetering et al. reported that cancer-derived organoids sensitivity to nutlin 3a is associated with the status of TP53 [5,37]. Saito et al. tested a library of 339 compounds in clinical use in biliary cancer PDO and observed 22 drugs that successfully inhibited the growth of organoids. Among these there were two antifungal compounds (amorolfine and fenticonazole), which suppressed iCCA organoids’ growth, while not causing significant toxicity to normal biliary epithelial cells [5]. Moreover, PDO can be used in animal models, injected subcutaneously or orthotopically, to generate PDO-derived PDX. Subcutaneous injection of CCA PDO in NSG mice have enabled the study of tet-on tet-off gene modulation in an in vivo model that maintains the same phenotype as of the human source tumour [32]. PDO generated from liver biopsies of colorectal cancer were shown to reproduce in the liver of NSG mice the same growth and drug response pattern observed in human [26]. Specifically, PDO from Regorafenib-sensitive patients showed a vessel co-option growth pattern, while PDO from Regorafenib-progressing patients manifested a desmoplastic pushing histopathological growth patterns in line with data observed in human patients [38]. This suggests that PDO may be able to reproduce the cancer-to-microenvironment interactions in vivo and that PDO-derived PDX could serve as an appropriate model to investigate mechanisms of resistance to agents targeting the microenvironment. Human CCA poses a remarkable challenge in the clinical oncological management. In an era where precision oncology is the central focus of oncological treatments, molecular characterization of advanced biliary cancers becomes one of the main challenges due to the following issues: 1) conversely to liver biopsies that usually provide enough tissue for molecular studies in iCCA, fine needle biopsies of pCCA and dCCA may pose some limitations for a mutational profile [39]. Nonetheless the positive experience of precision oncology in CCA has been mainly focused on iCCA with FGFR or IDH alterations (Moscato trial); 2) it is becoming more and more evident that phenotypic changes play an important role for the drug response, which adds to the information gathered from the somatic mutational profile. In order to perform a comprehensive molecular characterization at different levels (DNA, RNA and proteins) more tissue is needed, and single biopsies may not be sufficient. CCA PDO represent a useful tool to expand the epithelial cancer component and to allow the application of a wide range of different technologies that would not otherwise be possible. This application would also enable gathering information on advanced CCA, comparing how advanced CCA may differ and evolve from early stage resected CCA that have been characterised so far, and understanding the contribution of the molecular heterogeneity in cancer progression with regards to the different CCA subtypes. In addition, PDO offer the possibility to optimize and enhance precision oncology by underpinning a programme of individualized oncology. Recent evidence has shown that PDO can mimic ex vivo and in vivo responses observed in the patient opening the way to use PDO as a platform to guide therapeutic decisions [26]. PDO can be grown in a clinically meaningful time ranging between 6 and 10 weeks, maintain the same genotypic and phenotypic profile in the culture and has a positive and negative predictive value of 88% and 100%, respectively, in predicting the response in the patient [26]. Interestingly, Vlachogiannis et al. showed how the value of PDO in predicting the drug response goes beyond molecularly targeted drugs and can be applied to chemotherapy drugs or multityrosine kinases for which a molecular predictive biomarker has not been identified to date [26]. Information obtained through these approaches offer the possibility to have a genomic and drug-response profile for each patient and aid the selection of second line treatment by avoiding unnecessary toxicities and impacting on the quality of life of CCA patients.

## 6. Limitations of Patient Derived Organoids

Cancer PDO has shown to be a promising model to aid tumour biology understanding and precision oncology. However, there are still several open issues that need to be addressed before their use can be widely applied and their potential be implemented in clinical practice. We can foresee three main checkpoints in a successful PDO culture: 1) the establishment step with a PDO line growing within the first 2–4 weeks, 2) the ability to re-expand after splitting and 3) the capacity to grow back after cryopreservation. Several authors have shown that the efficacy rate of gastrointestinal cancer PDO to overcome all the three checkpoints is around 70% [26,40,41]. Unfortunately, the evidence for CCA PDO is still too sparse to be able to validate these findings. In our experience, PDO from highly chemoresistant CCA could grow in 5–6 weeks to a stage where functional analyses were possible [32]. Saito et al. reported a low efficiency rate, which seemed dependent on the subtype of biliary tumour (50% for iCCA and 20% for other biliary cancers). However, tumour PDO for which long term culture failed, did not harbour any driver gene mutations, raising the possibility that cultures were established from the contaminating non-cancerous cells [5]. In addition, some of the differences may be due to the early stage vs. advanced disease status. PDO success rate seems to be influenced by cellularity [4,26]; thus, PDO from extrahepatic biliary cancers were thought to be more challenging than iCCA. Unfortunately there is not enough data to confirm or refute this hypothesis; however Tiriac et al. have recently shown that the efficiency rate of PDO from pancreatic cancer fine needle biopsies is 70% [41]; given the diagnostic pathway of pancreatic cancer is very close to that of extrahepatic biliary cancers, it is sensible to speculate that access to tissues should not represent a limiting factor in pCCA and dCCA PDO. Cancer PDO reproduces 3D morphological structures that resemble the histological architecture of the source tumour. They comprise of cancer cells that have the ability to reshape within the ECM base and form glandular or tubular structures; however, they lack the microenvironment components and over time, despite maintaining their 3D morphology, they become pure epithelial cancer 3D cell lines. This may represent a remarkable limitation given the recent promise of therapies manipulating the tumour infiltrating immune cells. In order to overcome this challenge either co-cultures or air–liquid interphase (ALI) organoid models have been developed. Neal et al. generated ALI PDO from resected and metastatic tumours [42]. Continued growth >100 days did not always maintain complex tissue architecture suggesting that this method is more suitable for the purpose of a “live drug sensitivity platform” than long term disease modelling. Immune cells tended to decrease over 1 month of culture but could be preserved for longer with the addition of interleukin-2 [42]. Interestingly, the tumour infiltration lymphocytes within the PDO recapitulated the TCR repertoire of the original tumour, and preserved the ability to induce an antitumour immune response in plastic [42]. Likewise, co-cultures of cancer PDO with different components of the microenvironment have been successful. Dijkstra et al. co-cultured PDO prestimulated with Interferon γ with autologous T cells from peripheral blood lymphocytes [43]. Interestingly, they observed a tumour-specific T cell response when the co-cultures were established from mismatch deficient tumour PDO, while no activity was observed in the case of MHC class I deficient lines [43]. There is growing evidence showing that cancer is a heterogeneous disease with a number of clones that can outgrow and provide growth advantage over others. PDO derive from a core of tissue and, as such is subjected to sampling bias. PDO derived from multiple samples within the same tumour has confirmed that PDO recapitulate the intratumour heterogeneity in colorectal cancer [26]. Data on the intratumour heterogeneity of CCA is limited. Li et al. established 17 PDO lines from three resected patients with iCCA and reported that less than 30% of the variance in the drug response is due to intratumour heterogeneity [35]. Thus, more studies are needed to understand the level of intratumour heterogeneity of CCA and how much this issue can represent a limiting factor in biliary cancer. Recent efforts have tried to derive a protocol for the generation of PDO from circulating tumour cells (CTC) [44]. The efficiency rate reported so far is quite low and mainly positive in diseases with a high burden of CTC such as prostate cancers [25]. Moreover, CCA have shown to have a limited number of EpCam+ CTC, limiting this technology in its current form [45].

## 7. Conclusions

PDO are promising new tools to model CCA. They can mimic the cell-to-cell interaction, the cell-to-ECM interplay and the drug sensitivity of human cancers. More recent evidence suggests that co-cultures of PDO with various components of the microenvironment is feasible and help to establish mini organs on a chip that can better recapitulate the complexity of human disease. Multiple evidences suggest that PDO can be established from CCA patients. However, more studies are needed in order to understand the efficiency rate of CCA PDO in clinical practice, their differentiation according to the subtype and their potential clinical applicability.

## Figures and Tables

**Table 1:** Comparison of the main cancer models considering time, cost, establishment success rate, robustness, high throughput suitability and main limitations.

Comparison	2D Cancer Cell Lines (CCLs)	Tumour Spheroids	Patient-Derived Organoids (PDO)	Patient-Derived Xenografts (PDX)
Time	Days	Days	Weeks	Several Months
Cost	Low	Low	Medium	Expensive
Establishment success rate	Low: poor adaptation of patient-derived cells to in vitro culture conditions	High	Medium-High	Tumour type dependent
Robustness	Low: lacking of cell–cell interaction, cell–ECM interaction and absence of tumour microenvironment cause a misestimation in therapy prediction	Medium: recapitulate cell–cell interaction; more reliable model in therapy prediction compared to 2D cell lines	High: resemble morphologically, genomically and histologically the tissue of origin; reported as powerful preclinical in vitro model (with a high predictive value) to predict patient-specific therapy response	High: recapitulate the realistic tumour environment with the establishment of vasculature after engraftment; this allows to use PDX as in vivo preclinical models to evaluate patient-specific therapy response with a high predictive value
High throughput drug screening suitability	Suitable	Suitable	Suitable	Not suitable
Main limitations	Tend to accumulate mutations and to lose their identity with cell passages; do not recapitulate the microenvironment conditions observed in vivo (presence of stroma cells, immune system and blood vessels)	Do not recapitulate histological and morphological features of the tumour tissue; lack of original stroma and vasculature components	Lack of original stroma and vasculature components	Lack of a natural tumour microenvironment; presence of recipient stroma cells to the engraftment site; do not allow to reproduce crucial interactions (stroma-immune cells, immune cells-tumour)
